# Treatment of Alzheimer’s disease in Brazil: II. Behavioral and psychological symptoms of dementia

**DOI:** 10.1590/S1980-57642011DN05030006

**Published:** 2011

**Authors:** Francisco de Assis Carvalho do Vale, Ylmar Corrêa Neto, Paulo Henrique Ferreira Bertolucci, João Carlos Barbosa Machado, Delson José da Silva, Nasser Allam, Márcio Luiz Figueredo Balthazar, Group Recommendations in Alzheimer’s Disease and Vascular Dementia of the Brazilian Academy of Neurology

**Affiliations:** 1 Federal University of São Carlos (UFSCar), Department of Medicine (DMed), São Carlos SP, Brazil; 2 Federal University of Santa Catarina (UFSC), Department of Internal Medicine, Florianópolis SC, Brazil; 3 Federal University of São Paulo (UNIFESP), Sector of Behavioral Neurology - Escola Paulista de Medicina, São Paulo SP, Brazil; 4 Aurus IEPE – Institute of Research and Education on Aging of Belo Horizonte; Faculty of Medical Sciences of Minas Gerais (FCMMG), Department of Geriatric Medicine of Hospital Mater Dei, Belo Horizonte MG, Brazil; 5 Neurosciences Center of Hospital das Clinicas of the Federal University of Goiás (UFG). Integrated Institute of Neurosciences (IINEURO), Goiânia GO, Brazil; 6 University of Brasilia (UnB), Laboratory of Neurosciences and Behavior, Brasília DF, Brazil; 7 University of Campinas (UNICAMP), Faculty of Medical Sciences (FCM), Department of Neurology, Campinas SP, Brazil

**Keywords:** Alzheimer’s disease, dementia, behavioral and psychological symptoms of dementia, treatment

## Abstract

This article reports the recommendations of the Scientific Department of Cognitive Neurology and Aging of the Brazilian Academy of Neurology for the treatment of Alzheimer’s disease (AD) in Brazil, with special focus on behavioral and psychological symptoms of dementia (BPSD). It constitutes a revision and broadening of the 2005 guidelines based on a consensus involving researchers (physicians and non-physicians) in the field. The authors carried out a search of articles published since 2005 on the MEDLINE, LILACS and Cochrane Library databases. The search criteria were pharmacological and non-pharmacological treatment of the behavioral and psychological symptoms of AD. Studies retrieved were categorized into four classes, and evidence into four levels, based on the 2008 recommendations of the American Academy of Neurology. The recommendations on therapy are pertinent to the dementia phase of AD. Recommendations are proposed for the treatment of BPSD encompassing both pharmacological (including acetyl-cholinesterase inhibitors, memantine, neuroleptics, anti-depressives, benzodiazepines, anti-convulsants plus other drugs and substances) and non-pharmacological (including education-based interventions, physiotherapy, occupational therapy, music therapy, therapy using light, massage and art therapy) approaches. Recommendations for the treatment of cognitive disorders of AD symptoms are included in a separate article of this edition.

## Abstract (pt)

Esse texto apresenta as recomendações da Academia Brasileira de Neurologia, por intermédio do seu Departamento Científico de Neurologia Cognitiva e do Envelhecimento, para o tratamento da doença de Alzheimer (DA) no Brasil, enfocando os sintomas comportamentais e psicológicos da demência (SCPD). Trata-se de uma revisão ampliada das diretrizes publicadas em 2005, resultada de um consenso envolvendo pesquisadores da área, médicos e não médicos. Os autores realizaram uma busca de artigos publicados a partir de 2005 nas bases MEDLINE, LILACS e Cochrane Library. A busca foi direcionada para tratamento farmacológico e não farmacológico dos sintomas comportamentais e psicológicos da DA. Os estudos foram categorizados em quatro classes e as evidências em quatro níveis, com base nas recomendações da Academia Americana de Neurologia publicadas em 2008. As recomendações terapêuticas referem-se à fase demencial da DA. Apresentam-se recomendações para o tratamento dos SCPD, tanto farmacológico (incluindo inibidores da acetilcolinesterase, memantina, neurolépticos, antidepressivos, benzodiazepínicos, anticonvulsivantes e outros fármacos e substâncias), como não farmacológico (incluindo intervenções educacionais, fisioterapia, terapia ocupacional, musicoterapia, terapia com luz, massagem e arterapia). As recomendações para o tratamento dos transtornos cognitivos da DA são apresentadas em outro artigo desse fascículo.

## Introduction

In 2005, the Scientific Department of Cognitive Neurology and Aging (DCNCE-ABN) of the Brazilian Academy of Neurology published a set of recommendations and suggestions for the treatment of Alzheimer’s disease (AD).^1^ The present report comprises an updated version of these recommendations for treatment of behavioral and psychological symptoms of dementia (BPSD) based on current literature. The recommendations are part of a consensus effort involving a multi-disciplinary group of specialist researchers (physicians and non-physicians) also overseen by the DCNCE-ABN. Recommendations for the treatment of cognitive disorders of AD symptoms are included in a separate article of this edition.

The authors carried out a search of articles published since 2005 on the MEDLINE (PubMed), LILACS and Cochrane Library databases. The theme was split into two topics for the search:

(I) pharmacological treatment, including acetyl-cholinesterase inhibitors (AChEI), memantine, antipsychotics (neuroleptics), benzodiazepines, anti-convulsants, anti-depressives and other drugs (*Ginkgo biloba* extract, paracetamol, melatonin and testosterone); and
(II) non-pharmacological treatment including educational or psycho-educational interventions, rehabilitation/physical activity, occupational therapy, music therapy, physiotherapy, therapy using light, massage, art therapy and aromatherapy.

Studies retrieved were categorized into four classes, and evidence into four levels (See Table), based on the 2008 recommendations by the American Academy of Neurology.^2,3^. A draft of the recommendations was then presented to a panel of researchers from various disciplines (Neurology, Psychiatry, Geriatrics, Neuropsychology and Speech therapy) for discussion and consensus.

**Table:** Level of evidence.

**A.**	Established as effective, ineffective or prejudicial (or establish as useful/predictive or not useful/predictive) for a given condition in the specified population. (Classification level A requires at least two consistent Class I studies)*.
**B.**	Probably effective, ineffective, or prejudicial (and probably useful/predictive or not useful/predictive) for a given condition in the specified population. (Classification level B requires at least one consistent Class I or two Class II studies).
**C.**	Possibly effective, ineffective, or prejudicial (and probably useful/predictive or not useful/predictive) for a given condition in the specified population. (Classification level C requires at least one consistent Class II, or two Class III studies).
**U.**	Insufficient or conflicting data; based on current knowledge, the treatment (trial, prediction) is not proven.

* In exceptional cases, a convincing Class I study may suffice for A recommendation if: (1) all criteria are fulfilled, (2) the magnitude of the effect is large (relative degree of better result >5 and lower limit of confidence interval >2).

In April 2011, a work group from the American National Institute on Aging and the Alzheimer’s Association published recommendations for the diagnosis of dementia due to Alzheimer’s disease^4^ consisting of a revision of the diagnostic criteria for AD published in 1984.^5^ In the same period, the group also published recommendations for the diagnosis of mild cognitive impairment due to AD^6^ along with recommendations for application in the research setting containing criteria for the so-called “pre-clinical” stages of AD.^7^

The recommendations for treating AD proposed by the ABN apply to the dementia phase of the disease, whilst the present studies assessed were based on the definition of probable AD from the 1984 criteria.

This report is organized under two sections (pharmacological treatment and non-pharmacological treatment). With regard to the recommendations related to pharmacotherapy, it should be noted that these are based on scientific studies, whereas the prescribing physician must still check whether the drug is approved by the National Health Surveillance Agency (ANVISA).

## Pharmacological therapies

### Antipsychotics (neuroleptics)

The term “behavioral and psychological symptoms of dementia” (BPSD) is used to describe a set of non-cognitive symptoms which can manifest in dementia syndromes (e.g. depression, apathy, agitation, hyperactive behavior, sleep disturbances, anxiety, delirium and hallucinations). Identifying BPSD is important since they manifest in the majority of individuals with dementia during the course of the base disease (35-75% of patients).^8^

Individuals with AD have a greater number of comorbidities, with around 60% presenting three of more, resulting in the use of several medications.^9^ Drug interactions and polypharmacy may play a major role in the etiology of behavioral disorders seen in some patients with dementia.^10^ A multi-disciplinary team is key to proper management of polypharmacy and rational use of medications.^11,12^

One of the seminal and largest studies on efficacy of neuroleptics, the CATIE-AD, included 421 patients with AD and psychosis or with agitation/aggressive behavior. Patients were randomly assigned for treatment with a flexible dose of olanzapine, quetiapine, risperidone or placebo for up to 36 weeks. The patients were randomized for treatment with different medicines. Behavioral and psychiatric symptoms, functional abilities, cognition, care needs and quality of life were measured at regular intervals. In the descriptive analysis of the clinical results of these patients in terms of habitual care, some clinical symptoms improved following treatment with atypical anti-psychotics. Anti-psychotics are most effective for specific symptoms such as anger, aggressivity and paranoid ideas. Functional abilities, care needs, and quality of life do not appear to improve by treatment with antipsychotics.^13^

A thorough assessment is required encompassing clinical (e.g. infections, constipation, pain), psychiatric (e.g. depression, anxiety), environmental (e.g. ICU) and psychosocial (e.g. abandonment, aggression, change in environment) problems which could be related to the disorder. If possible, the underlying cause should be treated or modified prior to commencing medicamentous treatment, provided this does not pose a safety risk to the patient or caregivers. Thus, before commencing treatment with new medications, check whether the current clinical signs and symptoms are related to behavioral changes such as *delirium*, pain or an acute clinical condition (e.g. urinary infection, obstipation, pneumonia must be ruled out as a cause of the behavioral changes).^14^

Neuroleptics may have some utility as maintenance treatment of more severe neuro-psychiatric symptoms, but this benefit must be weighed against potential side effects. Anti-psychotic agents, when indicated, must be reassessed and risk/benefit considered, through continuous assessment.^15^ Monotherapy should first be given using low doses, which should then be steadily escalated until attaining the desired therapeutic effect, a process which may take several weeks.^16^ The antipsychotic should be reduced after behavioral symptoms have been controlled in order to determine whether the treatment is still needed.^17,18^

Upon resolution of BPSD, the antipsychotic can be withdrawn, and in most cases symptoms do not recur.^19-21^ Antipsychotics can have serious side effects such as elevated stroke risk, increased mortality, parkinsonism and cognitive disorders.^22-24^. Earlier recommendations by the American Academy of Neurology suggested the use of antipsychotics only after non-response to treatment with non-pharmacological approaches and optimization with anticholinesterasics and memantine.^20,25,26^

To summarize, considering the information currently available, antipsychotics have a role in treating more severe BPSD associated to dementia, such as *delirium* and hallucinations, intense agitation and aggressivity, but do not appear to improve functionality, reduce care needs, or improve quality of life in this patient group. After failure of non-pharmacological treatment as a first approach to manage these symptoms, and also use of selective serotonin reuptake inhibitors, anticonvulsants, anticholinesterases and memantine, the lack of safer alternative forces the use of antipsychotics to treat neuropsychiatric symptoms in dementia. Moreover, there is sufficient evidence favoring the use of atypical agents over typical agents, although no specific agent has yet been defined as the drug of choice in the available literature. There is an urgent need for new therapeutic options. Antipsychotic medications are linked to serious adverse events, including increased risk of death, strokes, tardive dyskinesia, malignant neuroleptic syndrome, hyperlipidemia, weight gain, diabetes mellitus, sedations, parkinsonism, and cognitive decline. There are no label indications for the use of neuroleptics in individuals with dementia. Patients and family members must be advised of the potential benefits and risks of antipsychotic agents, particularly mortality risk.

**Recommendations** – (1) Sufficient evidence exists to recommend antipsychotics for the treatment of psychotic symptoms in moderate to severe Alzheimer’s disease (Level B) and for the treatment of agitation and aggressivity (Level A), when other non-pharmacological approaches have failed to promote a response, and after ruling out any other mitigating factors. Treatment should be first administered at low doses, and only after risk/benefit assessment and full discussion with the patient (clinical conditions permitting), family members and caregivers; (2) Atypical neuroleptics should be preferred because they produce fewer side effects and do not pose any greater risk of stroke or death compared to conventional neuroleptics (Level B); (3) Few studies have investigated the repercussions of neuroleptic use beyond 12 weeks but considerable clinical experience supports the practice (Level U).

### Benzodiazepínes

Benzodiazepines and similar agents can be used for anxiety, insomnia, in cases of acute agitation with increased risk of falls, confusion, memory impairment, respiratory complications which in rare cases can lead to paradoxical disinhibition. Lorazepam and oxazepam, neither of which contains active metabolites, are preferable to agents with long half-lives such as diazepam or clonazepam.^16,21,27,28^

**Recommendations** – The few specific studies on BPSD in conjunction with data from the literature show modest benefits of benzodiazepine use, and despite a series of adverse effects, indicate that it has a role in the treatment of patients with acute anxiety, with infrequent episodes of agitation or those requiring sedation for a particular procedure such as dental treatment or a diagnostic exam (Level C).

### Acetylcholinesterase inhibitors (AChEI)

A meta-analysis of studies on the efficacy of cholinesterase in the treatment of BPSD in AD has shown a slight beneficial effect.^29^ Using total scores on the Neuropsychiatric Inventory, results of six Class I studies were pooled, assessing metrifonate (three trials on the currently unavailable drug), galantamine (two trials) and donepezil (one trial), giving a total of 2927 patients. The difference in favor of the cholinesterase inhibitors was 1.72 points (95% confidence interval, 0.87-2.57 points) from a possible score of 144 on the NPI.

A systematic review using total scores on the NPI identified four Class I studies of galantamine for the treatment of BPSD in AD.^30^ This beneficial effect was observed after 6 months at a dose of 16 mg/day with a difference in favor of galantamine versus placebo of 2.4 points (95% confidence interval, 0.32-3.84 points) in the cases observed, and 2.1 points (95% confidence interval, 0.16-4.04 points) in the intention to treat (ITT) cases. At a dose of 24mg/day, after 6 months of treatment, a difference was seen in favor of galantamine over placebo of 2.09 points (95% confidence interval, 0.34-3.84 points) in the cases observed. The difference in favor of galantamine was mainly due to poorer scores on the NPI among the placebo group (Class I study).^31^

A systematic review identified two Class I studies comparing rivastigmine against placebo, also using total score on the NPI, which reported no difference between the groups.^32^

A systematic review identified four Class I trials comparing donepezil with placebo, using total score on the NPI, which revealed a positive difference in 3 of the studies with a difference of 2.62 points (95% confidence interval 0.43-4.88 points) in favor of donepezil at 10 mg versus placebo for 24 weeks, and no difference in one study.^33^ Stratified assessments on the NPI identified improvement in specific domains: a Class I study showed a difference on depression/dysphoria and apathy/indifference domains^34,35^ while another found changes across all domains except elation/euphoria (Class I).^36^ A Class I trial specifically assessing a population with AD and agitation revealed no benefit from donepezil treatment on the NPI or the *Cohen-Mansfield Agitation Inventory* (CMAI).^37^

**Recommendations** – Study results are conflicting regarding the benefits of cholinesterase inhibitors in the treatment of BPSD of AD when assessed using global measures such as total NPI score (Level U). By contrast, there is evidence of benefit (Level A) for the treatment of specific symptoms including depression/dysphoria, anxiety and apathy/indifference. Good clinical practice guidelines recommend maximizing the cholinergic strategy in the management of BPSD in AD.

### Memantine

Pooled data from a systematic review of three Class I studies on the efficacy of memantine for controlling BPSD in moderate to severe AD revealed benefits of the drug evidenced by a 2.76 point difference on the NPI (95% confidence interval, 0.88-4.63 points).^38^ This benefit was largely due to worse scores by the placebo group.^39^ Stratified assessment of the NPI showed benefits for agitation/aggression, irritability/lability and nighttime behavior domains.^40^ The evidence indicated that these manifestations occurred less frequently in the treated group, but not that memantine improved preexisting conditions.^38^ This effect was not however, observed In patients with mild to moderate AD.^38^

**Recommendations** – The use of memantine in patients with moderate to severe AD probably reduces manifestation of some BPSD (Level B).

### Anti-convulsants

A literature review identified seven randomized studies of anticonvulsants for the treatment of BPSD in demented individuals. Two of these used carbamazepine and five valproic acid.^41^ None of the studies with valproic acid showed any benefit while a small study involving 14 patients actually reported a worsening of agitation/aggression domains on the NPI. Of the carbamazepine studies, one showed no benefits while the other showed improvement on the *Brief Psychiatric Rating Scale*, although the treated group had more advanced disease than the placebo group, having longer time with the disease (4.0±5.1versus 2.8±2.8 years) and lower Mini Mental State Exam scores (3.9±6.2versus 8.3±7.2points), where these disparities compromise evaluation of the result.^42^

**Recommendations** – The results of the studies assessed remain controversial and are insufficient to indicate the use of anticonvulsants for the treatment of BPSD in AD (Level U).

### Anti-depressives

A systematic review identified five Class I trials of serotonin reuptake inhibitors in the treatment of agitation in demented individuals. The corresponding meta-analysis revealed benefits on the CMAI (0.89 point difference with 95% confidence interval between 0.57-1.22).^43^ Based on total NPI scores, a Class I study^44^ and two class II studies^45,46^ showed no benefit from sertraline, whereas the study by Finkel et al.^46^ observed improvement in a subgroup of the NPI including dysphoria, irritability, anxiety and agitation/aggressivity in moderate to advanced AD with BPDS (60% improvement in treatment versus 40% in placebo group, p=0.006). One Class II study showed benefits from citalopram in the treatment of irritability and depressive mood in individuals with dementia^47^ whereas another Class II study evidenced benefit from citalopram in the improvement of agitation/aggression and lability in demented patients with BPSD on the *Neurobehavioral Rating Scale*.^48^ Studies with no placebo group comparing trazodone with haloperidol using the CMAI,^49^ and escitalopram with risperidone on the NPI,^50^ found similar results for alleviation of BPSD.

The treatment of depression as a comorbidity in AD was not assessed in this study.

**Recommendations** – The use of anti-depressives may possibly be beneficial in the treatment of some BPSD in AD (Level C).

### Other drugs and substances

Class II studies of paracetamol,^51^ testosterone,^52^ and melatonin^53^ showed no benefit, while a Class II study of *Ginkgo biloba* extract EGb 761^54^ and one on latrepirdine,^55^ found a favorable difference in total NPI scores among the treated groups.

**Recommendations** – The currently available evidence does not allow the recommendation of paracetamol, testosterone, melatonin or *Ginkgo biloba* for the treatment of BPSD in AD (Level U).

## Non-pharmacological therapies

There is growing interest among researchers in studies involving several forms of non-pharmacological interventions in search of greater levels of evidence through randomized controlled trials. However, many such studies are limited by small samples and the absence of control groups. The investigations also tend to have flawed methodological approaches in as far as they lack in-depth description of the procedures adopted to perform the study. These factors can lead to inconsistent data which in turn limits the reliability of results. However, these drawbacks should not prevent the indication of these treatments, given that studies with reliable levels of evidence have shown good results both in terms of statistical significance and in routine clinical practice.

### Education-based interventions

Randomized controlled trials have shown that educational programs improve BPSD in patients, distress in caregivers, and delay institutionalization while often dispensing with the need to use medications. Moreover, a significantly improved response by caregivers to the behavioral and aggressivity disorders of patients has been noted, along with reduced frequency of these disorders in this patient group. Persistent improvements in depression and agitation have been noted among patients and caregivers as a result of education-based programs and behavioral strategies.^56,57^

### Physiotherapy

Randomized controlled trials have revealed that motor rehabilitation exercises with physical activity and supervised programs of regular exercise can reduce BPSD in patients, as well improve cognition and mood. However, further studies are needed to confirm these findings.^58,59^

### Occupational therapy (OT)

A randomized, controlled trial entailing 10 sessions of OT over 5 weeks was found to promote functional, clinical and behavioral improvement in individuals with dementia, and consequent improvements in quality of life among both patients and caregivers. Patients with severe BPSD however, were excluded from the cited study.^60^ Another study demonstrated improved apathy following OT using a combination of psychomotor activity and music and art therapies.^61^

### Music therapy

Recently, an increasing number of randomized controlled trials based on music therapy have been conducted, showing efficacy in managing BPSD in moderate to severe AD.^62^ Improvements were also seen in depression and apathy, particularly in patients with mild to moderate AD.^63^

### Other treatment approaches

Therapy using light, massage, aromatherapy, art therapy among other activities, although demonstrating some degree of effectiveness in a number of trials, lack randomized, controlled evidence-based studies to confirm significant results.

**Recommendations** – (1) Non-pharmacological strategies may be used for the treatment of BPSD in AD. Educational interventions are recommended (Level B) as well as other treatment strategies including Physiotherapy (Level C), Occupational Therapy (Level C) and Music therapy (Level C). (2) Insufficient evidence is available to recommend light therapy, massage, aromatherapy or art therapy for treating BPSD in AD (Level U).

## GROUP RECOMMENDATIONS IN ALZHEIMER’S DISEASE AND VASCULAR DEMENTIA OF THE BRAZILIAN ACADEMY OF NEUROLOGY

**Amauri B. da Silva** [UNINEURO, Recife (PE)]; **Ana Cláudia Ferraz** [Serviço de Neurologia do Hospital Santa Marcelina (SP)]; **Analuiza Camozzato de Pádua** [Universidade Federal de Ciências da Saúde de Porto Alegre (UFCSPA); Hospital de Clínicas de Porto Alegre (UFRGS) (RS)]; **Antonio Lúcio Teixeira** [Departamento de Clínica Médica, Faculdade de Medicina da Universidade Federal de Minas Gerais, Belo Horizonte (MG)]; **Ayrton Roberto Massaro** [Instituto de Reabilitação Lucy Montoro (SP)]; **Benito Pereira Damasceno** [Departamento de Neurologia da Universidade Estadual de Campinas (SP)]; **Carla Tocquer** [Universidade Federal do Rio de Janeiro (RJ)]; **Carlos Alberto Buchpiguel** [Departamento de Radiologia, Faculdade de Medicina da Universidade de São Paulo (SP)]; **Cássio Machado C. Bottino** [Programa Terceira Idade, Instituto de Psiquiatria do Hospital das Clínicas da Faculdade de Medicina da Universidade de São Paulo (FMUSP) (SP)]; **Charles André** [Faculdade de Medicina - UFRJ; SINAPSE Reabilitação e Neurofisiologia (RJ)]; **Cláudia C. Godinho** [Serviço de Neurologia do Hospital de Clínicas de Porto Alegre, Universidade Federal do Rio Grande do Sul (RS)]; **Cláudia Sellitto Porto** [Grupo de Neurologia Cognitiva e do Comportamento da Faculdade de Medicina da USP (SP)]; **Denise Madeira Moreira** [Departamento de Radiologia Faculdade de Medicina - UFRJ; Setor de Radiologia - INDC - UFRJ (RJ)]; **Eliasz Engelhardt** [Setor de Neurologia Cognitiva e do Comportamento - INDC - CDA/IPUB - UFRJ (RJ)]; **Elza Dias-Tosta** [Presidente da Academia Brasileira de Neurologia, Hospital de Base do Distrito Federal (DF)]; **Emílio Herrera Junior** [Departamento de Medicina Interna, Faculdade de Medicina de Catanduva (SP)]; **Gabriel R. de Freitas** [Instituto D’or de Pesquisa e Ensino; Universidade Federal Fluminense (RJ)]; **Hae Won Lee** [Instituto de Radiologia, Hospital das Clínicas da Faculdade de Medicina da Universidade de São Paulo e Hospital Sírio-Libanês (SP)]; **Ivan Hideyo Okamoto** [Departamento de Neurologia e Neurocirurgia; Instituto da Memória - Universidade Federal de São Paulo - UNIFESP (SP)]; **Jerusa Smid** [Grupo de Neurologia Cognitiva e do Comportamento do Hospital das Clínicas da Faculdade de Medicina da Universidade de São Paulo (FMUSP) (SP)]; **José Antonio Livramento** [Laboratório de Investigação Médica (LIM) 15, Faculdade de Medicina da Universidade de São Paulo (SP)]; **José Luiz de Sá Cavalcanti** [Departamento de Neurologia - INDC - UFRJ; Setor de Neurologia Cognitiva e do Comportamento - INDC - UFRJ (RJ)]; **Letícia Lessa Mansur** [Grupo de Neurologia Cognitiva e do Comportamento do Departamento de Neurologia da FMUSP; Departamento de Fisioterapia, Fonoaudiologia e Terapia Ocupacional da Faculdade de Medicina da USP (SP)]; **Liana Lisboa Fernandez** [Departamento de Ciências Básicas da Saúde, Fundação Universidade Federal de Ciências da Saúde de Porto Alegre (RS)]; **Márcia Lorena Fagundes Chaves** [Serviço de Neurologia do Hospital de Clínicas de Porto Alegre, Universidade Federal do Rio Grande do Sul (RS)]; **Márcia Radanovic** [Laboratório de Neurociências - LIM27, Departamento e Instituto de Psiquiatria da Faculdade de Medicina da Universidade de São Paulo (FMUSP) (SP)]; **Maria Teresa Carthery-Goulart** [Grupo de Neurologia Cognitiva e do Comportamento do Departamento de Neurologia da Faculdade de Medicina da USP; Centro de Matemática, Computação e Cognição, Universidade Federal do ABC (SP)]; **Mônica S. Yassuda** [Grupo de Neurologia Cognitiva e do Comportamento do Departamento de Neurologia da Faculdade de Medicina da USP; Departamento de Gerontologia, Escola de Artes, Ciências e Humanidades da USP (EACH/USP Leste) (SP)]; **Norberto Anízio Ferreira Frota** [Universidade de Fortaleza (UNIFOR), Serviço de Neurologia do Hospital Geral de Fortaleza (HGF) (CE)]; **Orestes Forlenza** [Laboratório de Neurociências - LIM27, Departamento e Instituto de Psiquiatria da Faculdade de Medicina da Universidade de São Paulo (FMUSP) (SP)]; **Paulo Caramelli** [Departamento de Clínica Médica, Faculdade de Medicina da Universidade Federal de Minas Gerais, Belo Horizonte (MG)]; **Regina Miksian Magaldi** [Serviço de Geriatria do Hospital das Clínicas da FMUSP, Centro de Referência em Distúrbios Cognitivos (CEREDIC) da FMUSP (SP)]; **Renata Areza-Fegyveres** [Grupo de Neurologia Cognitiva e do Comportamento do Hospital das Clínicas da Faculdade de Medicina da Universidade de São Paulo (FMUSP) (SP)]; **Renato Anghinah** [Grupo de Neurologia Cognitiva e do Comportamento do Hospital das Clínicas da Faculdade de Medicina da Universidade de São Paulo (FMUSP); Centro de Referência em Distúrbios Cognitivos (CEREDIC) da FMUSP (SP)]; **Ricardo Nitrini** [Grupo de Neurologia Cognitiva e do Comportamento do Hospital das Clínicas da Faculdade de Medicina da Universidade de São Paulo (FMUSP); Centro de Referência em Distúrbios Cognitivos (CEREDIC) da FMUSP (SP)]; **Rodrigo Rizek Schultz** [Setor de Neurologia do Comportamento do Departamento de Neurologia e Neurocirurgia da Universidade Federal de São Paulo, Núcleo de Envelhecimento Cerebral (NUDEC) - Instituto da Memória (UNIFESP) (SP)]; **Rogério Beato** [Grupo de Pesquisa em Neurologia Cognitiva e do Comportamento, Departamento de Medicina Interna, Faculdade de Medicina, UFMG (MG)]; **Sonia Maria Dozzi Brucki** [Grupo de Neurologia Cognitiva e do Comportamento da Faculdade de Medicina da Universidade de São Paulo; Centro de Referência em Distúrbios Cognitivos (CEREDIC) da FMUSP; Hospital Santa Marcelina (SP)]; **Tânia Novaretti** [Faculdade de Filosofia e Ciências, Campus de Marília, da Universidade Estadual Paulista (UNESP) (SP)]; **Valéria Santoro Bahia** [Grupo de Neurologia Cognitiva e do Comportamento do Hospital das Clínicas da Faculdade de Medicina da Universidade de São Paulo (FMUSP) (SP)];
